# Measurement of myocardial blood flow response to the cold pressor test with myocardial perfusion CMR

**DOI:** 10.1186/1532-429X-13-S1-P312

**Published:** 2011-02-02

**Authors:** Timothy A Fairbairn, Adam Mather, Adbul Larghat, John Greenwood, Sven Plein

**Affiliations:** 1 University of Leeds, Leeds, UK

## Objective

To measure Cold Pressor Test (CPT)-induced changes in MBF and compare them to adenosine induced (endothelial-independent) changes in MBF.

## Background

The CPT induces endothelial-dependent vasodilation with increased myocardial blood flow (MBF) in normal coronary arteries. It has been used to demonstrate abnormal coronary vasomotion by invasive (angiography) and non-invasive techniques (SPECT and PET). Cardiovascular Magnetic Resonance (CMR) allows the assessment of MBF in separate myocardial layers due to its high spatial resolution, but has not previously been used to measure physiological responses to CPT.

## Methods

Eleven healthy volunteers (age 23±5.4, 64% male) attended for a CMR perfusion scan (Phillips Intera 1.5T, 0.05mmol/kg Gd-DTPA, spatial resolution 2.3 x 2.3mm), performed at rest, during CPT (120s of foot immersion in 0-4°C water) and adenosine hyperaemia (140mcg/kg/min for 4 minutes). Each perfusion scan was separated by 15 minutes. Heart rate (HR) and blood pressure (BP) were simultaneously recorded .This information was then used to calculate mean arterial pressure (MAP= 2*Diastolic BP (DBP)+ Systolic BP (SBP)/3), rate-pressure product (RPP= HR x SBP) and coronary vascular resistance (CVR= MAP/ MBF). MBF (ml/g/min) was estimated for a mid-ventricular slice by Fermi-constrained deconvolution.

## Results

**Figure 1:**
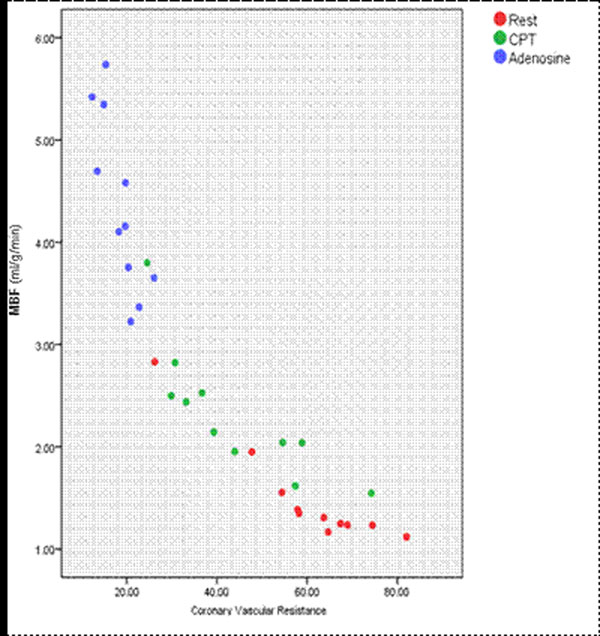
A scatter plot demonstrating the negative relationship between Coronary Vascular Resistance (CVR) and Myocardial Blood Flow (MBF, ml/g/min) for each perfusion examination.

MBF increased significantly from rest to CPT (1.5±0.5 to 2.3±0.6 ml/g/min, p=0.004) and from CPT to stress (4.4±0.8, p<0.001) (Table 1). Endocardial MBF was significantly higher than epicardial MBF at rest (p<0.001) and during CPT (p=0.008), however during adenosine hyperaemia epicardial MBF was higher (p=0.043). Regression analysis of haemodynamic factors identified Coronary Vascular Resistance (CVR) as the only independent predictor of MBF during rest, CPT and adenosine, (Figure 1).

**Table 1:** Haemodynamic parameters and Myocardial Blood Flow (ml/g/min), measured at rest, peak cold pressor test (CPT) and adenosine stress

	REST	P value	CPT	P value	ADENOSINE
**Heart Rate (bpm)**	70 ±9	0.001	84 ±12	0.02	97 ±12
**Systolic Blood Pressure (mmHg)**	120 ±10	0.025	132 ±16	0.002	114 ±14
**Diastolic Blood Pressure (mmHg)**	66 ±7	0.001	78 ±9	<0.001	60 ±9
**Mean Arterial Pressure (mmHg)**	84 ±6	0.022	94 ±15	<0.001	78 ±10
**Rate Pressure Product (mmHg/min)**	8441 ±1198	<0.001	11157 ±2018	0.972	11184 ±2031
**Coronary Vascular Resistance (mmHg/ml min g)**	60.5 ±14.8	0.01	43.9 ±15.4	<0.001	18.5 ±4.2
**Myocardial Blood Flow (ml/g/min)**	1.48 ±0.15	0.004	2.31 ±0.18	<0.001	4.36 ±0.25
**Endocardial Blood Flow (ml/g/min)**	1.7 ±0.6	NA	2.38 ±0.5	NA	4.09 ±0.7
**Epicardial Blood Flow (ml/g/min)**	1.38 ±0.5	NA	2.09 ±0.7	NA	4.53 ±1.1

## Discussion

Perfusion-CMR permits assessment of endothelial-dependent (CPT) and endothelial-independent (adenosine) MBF in a single examination. Furthermore, CMR demonstrates differences in the physiological response to CPT and maximal hyperaemia between the endocardium and epicardium. Future studies should establish the role of this new method in at risk groups such as those with diabetes or smokers.

